# Gretl—variation GRaph Evaluation TooLkit

**DOI:** 10.1093/bioinformatics/btae755

**Published:** 2024-12-24

**Authors:** Sebastian Vorbrugg, Ilja Bezrukov, Zhigui Bao, Detlef Weigel

**Affiliations:** Department of Molecular Biology, Max Planck Institute for Biology Tübingen, 72076 Tübingen, Germany; Department of Molecular Biology, Max Planck Institute for Biology Tübingen, 72076 Tübingen, Germany; Department of Molecular Biology, Max Planck Institute for Biology Tübingen, 72076 Tübingen, Germany; Department of Molecular Biology, Max Planck Institute for Biology Tübingen, 72076 Tübingen, Germany

## Abstract

**Motivation:**

As genome graphs are powerful data structures for representing the genetic diversity within populations, they can help identify genomic variations that traditional linear references miss, but their complexity and size makes the analysis of genome graphs challenging. We sought to develop a genome graph analysis tool that helps these analyses to become more accessible by addressing the limitations of existing tools. Specifically, we improve scalability and user-friendliness, and we provide many new statistics tailored to variation graphs for graph evaluation, including sample-specific features.

**Results:**

We developed an efficient, comprehensive, and integrated tool, *gretl*, to analyze genome graphs and gain insights into their structure and composition by providing a wide range of statistics. *gretl* can be utilized to evaluate different graphs, compare the output of graph construction pipelines with different parameters, as well as perform an in-depth analysis of individual graphs, including sample-specific analysis. With the assistance of *gretl*, novel patterns of genetic variation and potential regions of interest can be identified, for later, more detailed inspection. We demonstrate that *gretl* outperforms other tools in terms of speed, particularly for larger genome graphs.

**Availability and implementation:**

Commented Rust source code and documentation is available under MIT license at https://github.com/MoinSebi/gretl together with Python scripts and step-by-step usage examples. The package is available at Bioconda for easy installation.

## 1 Introduction

Advances in short-read-based resequencing have greatly improved our understanding of genomic variation in many different species (The 1000 Genomes Project Consortium *et al.* 2015, 1001 Genomes Consortium 2016, Peter *et al.* 2018). More recently, long reads have made it possible to assemble complete genomes with remarkable speed and precision. Moving from variant inventories to complete genomes facilitates much more comprehensive analysis and genome-wide comparison between samples. As an example, in the plant *Arabidopsis thaliana*, the level of detail provided by (nearly) complete genomes has already led to new insights into conservation of synteny and to much more accurate description of single-nucleotide polymorphisms, copy number variants (CNVs), and structural rearrangements (Goel *et al.* 2019, Jiao and Schneeberger 2020).

To mitigate the biases associated with a single reference genome, pan-genomes built from diverse sample collections are being created from increasingly complex genomes (Shang *et al.* 2022, Liao *et al.* 2023). A crucial tool for efficient storage and comprehensive analysis of genetic variations within diverse and intricate genomic regions is the variation graph, which condenses similar sequences into nodes and captures variations in a reference-free manner. Graph shape and structure depend on the choice of construction method and parameter set, requiring tuning and adjustment based on the genome complexity (Leonard *et al.* 2023) and the research question, highlighting the need for a comprehensive evaluation tool.

Genome graphs are typically stored in GFA (Graphical Fragment Assembly) format, a standardized data format, which is also the main input for *gretl*, the tool introduced here. Nodes in the graph represent DNA segments, connected by edges and each node has an associated DNA sequence and a unique identifier. GFA can store additional information like allele frequency, quality scores, or annotations, if needed. The format ensures interoperability among software tools, facilitating collaboration and analysis development. *gretl* fully supports GFAv1 files (http://gfa-spec.github.io/GFA-spec/GFA1.html), ensuring interoperability across a wide range of graph tools. Adopting GFAv2 is an option for the future, as more upstream graph tools migrate to GFAv2.

Several tools for genome graph analysis are available and being actively developed, including *odgi* (Guarracino *et al.* 2022), *vg* (Garrison *et al.* 2018), and *gfastats* (Formenti *et al.* 2022). While *odgi* and *vg* offer powerful platforms for modifying and analyzing genome graphs, there is still a need for tools that can rapidly compute an overview of a large number of statistical features for evaluation of variation graphs. Although *gfastats* is designed for statistics, its primary focus lies in assembly graphs, which have, in comparison with whole-genome graphs, distinct characteristics. While it does provide several useful statistics for genome graphs, its main function remains an overall toolkit for modifying GFA files and delivering high quality single individual genomes. In our benchmarking and comparison between the different methods, we excluded *gfastats*, because the run did not finish within a reasonable amount of time.

**Figure 1.**
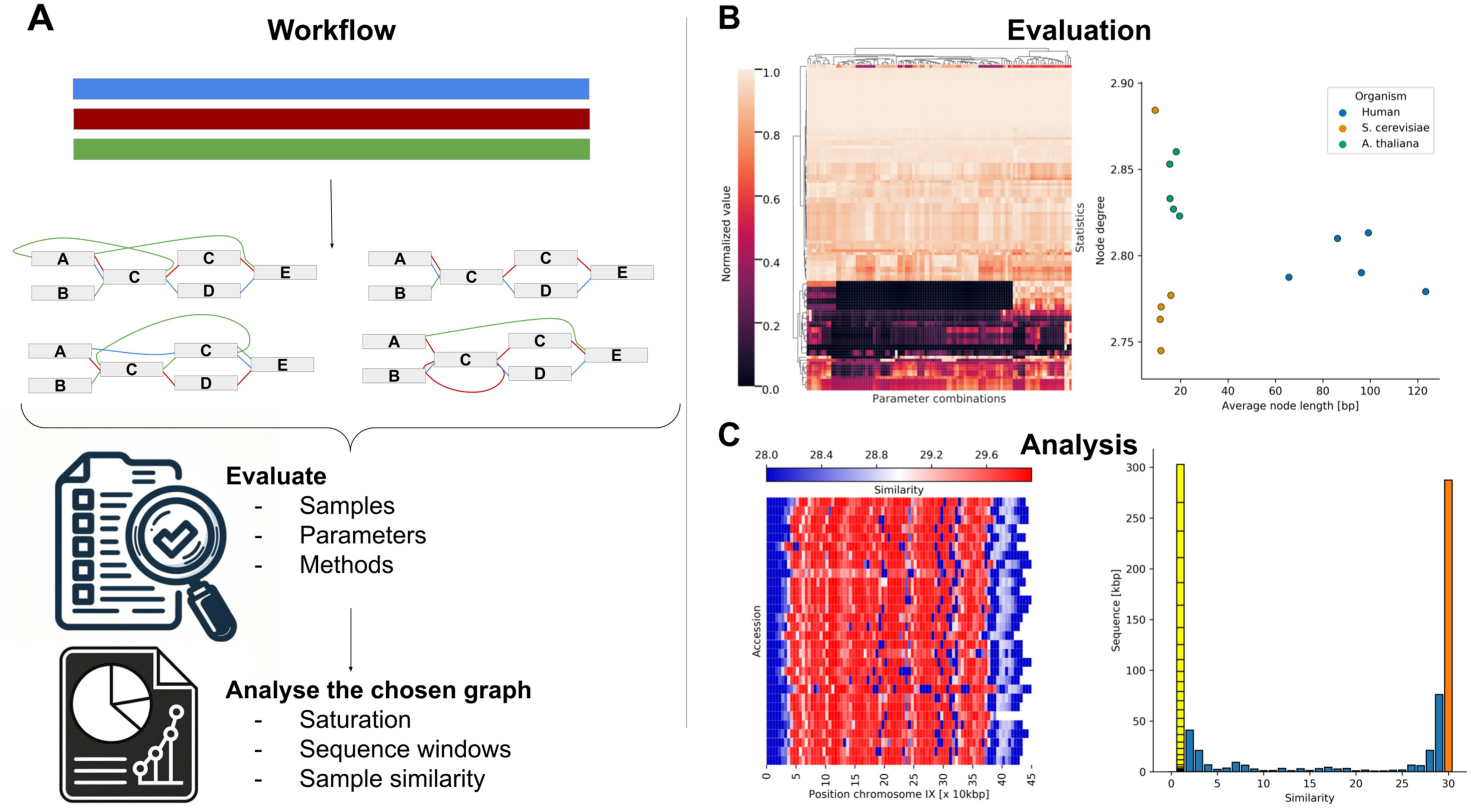
Gretl overview. (A) Genome graph construction workflow: genome graph properties are influenced by various factors, including parameter selection, sample curation, and methodology, all of which impact the layout and structure of the resulting genome graph. For evaluation purposes, multiple graphs can be simultaneously generated and compared to identify an optimal representation for a specific task. The selected graph can then be analyzed with *gretl*. (B) Visualization of *gretl* output: left, graphs can be clustered based on multiple statistics, grouping similar species or construction parameters (shown here, with normalized values). Right, scatter plot depicting two selected statistics across various graphs, facilitating comparisons between different species. (C) In-depth analysis of a selected genome graph (example from yeast): left, path-centric sliding window analysis of the *Saccharomyces cerevisiae* genome graph, highlighting regions of high similarity. Right, pan-genomic analysis of the genome graph. Sequences found only in a single sample are separated and each block represents one path of the graph.

One of the primary motivations behind our work was to provide a fast and efficient tool for the initial evaluation of newly constructed graphs. Building genome graphs is a complex process, and one often needs to rapidly assess their quality. *gretl* aims to address this need by offering an all-in-one tool that evaluates graph structure and composition (Fig. 1A).

With *gretl*, researchers can evaluate the graph using a variety of quantitative metrics and identify potential areas that require further investigation or refinement. As an example, graphs with high average depth are most likely highly collapsed, merging duplicated segments such as transposable elements (TEs) into a single structure, which in turn makes it harder to align sequences or sequencing reads to graphs, but it helps to understand the nature of transposed DNA segments or CNV. Moreover, one can generate statistics on genome-growth, pan-genome distribution and/or for specific paths (Supplementary Figs S1 and S6). This preliminary information can guide subsequent analyses and describe the properties of different species pangenomes, providing a solid foundation for further investigations.

## 2 Results

*gretl* offers valuable insights into genome graphs constructed using PGGB (Garrison *et al.* 2024) and Minigraph-Cactus (Hickey *et al.* 2024), as well as other graphs in GFA format. The only requirement is the availability of numeric node IDs, which can, if not already present, be converted from non-numeric node IDs via the *gretl node2int* subcommand. *gretl* provides several subcommands that offer comprehensive graph analysis, covering aspects such as graph complexity, interconnectedness, and node degree. We provide Python scripts and follow-along markdowns that can be used for post-processing and visualization of the output similar to the plots shown in the figures here, allowing further exploration and interpretation of the results.

To illustrate the capabilities of *gretl* for graph evaluation, we used pan-genome graphs constructed from chromosomes of *Homo sapiens* (*n* = 48 for Chr 14, 18, 19, 21, 22), *Saccharomyces cerevisiae* (*n* = 30 for Chr 1, 3, 5, 9, 10), and *A. thaliana* (*n* = 67 for Chr 1–5). Details about the datasets (Liao *et al.* 2023, O’Donnell *et al.* 2023, Wlodzimierz *et al.* 2023) and graph construction are given in Supplementary Data.

Firstly, the provided statistics enable the evaluation of graphs built with different parameters from the same dataset (Fig. 1B). Statistics generated by *gretl* (Supplementary Figs S1 and S6) can guide subsequent analyses and describe the properties of different species pangenomes.

Additionally, these values facilitate the evaluation and comparison of graphs from different methods or organisms, enabling insights into the complexity and structure of the genome (Supplementary Tables S1 and S7). It is important to note that some of these statistics may exhibit similar behavior and display high correlation due to their interconnected nature (Supplementary Fig. S5). Researchers can explore the impact of varying parameters during graph construction on the same dataset or analyze different species by comparing or clustering their genome graphs by statistical features (Fig. 1B).

In general, *gretl* offers more comprehensive information about the graph than other tools. The tabular output format provides an easy overview as well as seamless integration with scripting languages such as R and Python for post-processing.

Secondly, *gretl* facilitates in-depth comparison of specific graphs using a wide range of metrics. This analysis can be performed at both the graph level and the path level, providing researchers with comprehensive insights. At the graph level, various metrics and statistics can be explored to identify regions of interest, which can be further investigated in subsequent studies (Fig. 1C, Supplementary Figs S10 and S11). Sliding window analyses on sequence or node level give powerful insight into local complexity or distant sequence similarities (Supplementary Fig. S8). This could be, for example, useful to demonstrate the local complexity of possible QTL hits (Milia *et al.* 2024).

The path-centric analyses allow for computation of independent statistics and metrics for specific paths within the graph (Supplementary Table S3). This approach enables comparisons between different samples or populations, helping in the identification of path-specific differences within the pan-genome (Fig. 1C, Supplementary Fig. S4). Furthermore, it enables the identification of samples that display isolated or otherwise distinctive representations in the graph (Supplementary Fig. S3). By carefully examining the paths within the graph, researchers can uncover structural patterns, variations, and potential functional significance embedded within the genome. This comprehensive analysis of paths should contribute to a deeper understanding of the genome’s complexities and provide valuable insights for further research. A table of reported statistics including the name, description, and availability in other tools can be found in Supplementary Tables S2 and S3.

During testing on a 3.2 GHz AMD Epyc 64 core machine using chromosome 19 of the PGGB-built HPRC graph (Liao *et al.* 2023), which consisted of 48 samples (96 haplotypes, 1072 paths) with 3.02 million nodes and 4.21 million edges, our evaluation tool demonstrated a level of performance that should greatly encourage its adoption for any genome graph construction workflow. It computed simple summary statistics from GFA files twice as fast as other approaches in under five minutes, utilizing 2.91 GB of memory (Supplementary Fig. S2). We observed almost linear scaling properties (Supplementary Fig. S9).

## 3 Discussion

We contribute *gretl*, a fast, efficient, and user-friendly stand-alone tool for generating a wide range of statistics and insights into the structure and composition of genome graphs, complemented with a set of user-friendly Python scripts for downstream analyses. *gretl* generates 108 different metrics for a single variation graph. We highlight path-centric statistics and analyses especially designed for genome graphs that have not yet been implemented by other tools.

It is important to note that the quality of the genome assemblies used to generate the genome graph can significantly affect the accuracy and completeness of the generated metrics and subsequent downstream analyses. As such, it is essential to carefully evaluate and validate assembly quality before using *gretl*. In our experience, the building of graphs from complex genomes such as those of plants is highly affected by parameter choice.

While *gretl* can process any graph which adheres to the GFAv1 specification, it is required that node IDs are numeric, and a sorted ID space is necessary for all “Jump”-related statistics. We recommend using path-guided 1D SGD ordering, which can be achieved effectively using the “odgi sort-Y” functionality during the preprocessing stage (Guarracino *et al.* 2022, Heumos *et al.* 2024).

*gretl* is unique in that it provides both graph-based and path-based statistics, allowing users to gain insights into both the overall structure of the genome graph and the specific paths/samples through the graph that correspond to genetic variation. Finally, *gretl* is designed to be modular and extensible, allowing for the future addition of new features and statistics.

## Supplementary Material

btae755_Supplementary_Data

## Data Availability

Source code and documentation is available under MIT license at https://github.com/MoinSebi/gretl.
