# Supplementary material for: Gretl—variation GRaph Evaluation TooLkit
Source: Bioinformatics. 2024 Dec 24;41(1):btae755. doi: 10.1093/bioinformatics/btae755 (PMC11729725; doi:10.1093/bioinformatics/btae755)
Supplement: btae755_Supplementary_Data [file btae755_supplementary_data.pdf]

Gretl - Variation **GR**aph **E**valuation **T**oo**L**kit

Supplementary Information

Sebastian Vorbrugg, Ilja Bezrukov, Zhigui Bao, Detlef Weigel

1. Implementation **2**
2. Datasets **2**
3. Building and visualizing a pangenome **2**
4. Pangenome classification **2**
5. Relationship between samples and path/walks **2**
6. More detail information for some statistics **3**
7. Table of all reported features (gretl stats) **3**
8. Comparison with other tools **3**
9. Supplementary tables and figures **4**

1. Implementation

gretl has been implemented in the Rust programming language. It incorporates several Rust crates to enhance performance and enable multithreading. Furthermore, a new GFA format reader is provided as a library. In addition to handling the GFAv1 format utilized by ***gretl***, this reader can interpret GFAv2 as well and it can be used by other tools.

2. Datasets

Arabidopsis thaliana genomes were from (Wlodzimierz *et al.*, 2023), while *S. cerevisiae* genomes were from (O'Donnell *et al.,* 2023). An available PGGB-built human genome graph (Liao *et al.*, 2023), https://github.com/human-pangenomics/hpp_pangenome_resources; was used. Chromosomes 14, 18, 19, 21, 22 were downloaded from https://s3-us-west-2.amazonaws.com/human-pangenomics/index.html?prefix=pangenomes/freeze/freeze1/pggb/chroms/

3. Building and visualizing a pangenome

The *A. thaliana* and *S. cerevisiae* graphs were constructed using the PGGB pipeline (Garrison *et al.*, 2023) for each chromosome individually. We used the following parameter to construct the *S. cerevisiae* graph for the species comparison experiment: -p 90; -k 31, -n 30 -s 5000 -asm10. *Arabidopsis thaliana* graphs were constructed with the following parameters: -p 90 -s 10000 -G 2000 -n 67 -t 32 -k 49 -P asm5 -O 0.001 -G 700,900,1000. Parameter comparisons were conducted using *S. cerevisiae* genomes with various parameter combinations. The parameters we modified included -s (2k, 5k, 10k), -p (80, 90, 95), -k (19, 31), -n (15, 30, 60), and -P (asm5, asm20). The ***pggb*** workflow was executed with ***wfmash*** (v0.10.2-2-gb310bd1), ***seqwish*** (v0.7.8-3-gd9e7ab5), ***odgi*** (v0.8.2-92-gbfae0b3), and ***smoothxg*** (v0.6.8-31-g06bbf35).

We used BandageNG (https://github.com/asl/BandageNG; version: v2022.09), a fork of the original Bandage (https://github.com/rrwick/Bandage), for visualization of variation graphs.

4. Pangenome classification

We utilized the characteristics of the graph to classify different levels of relatedness for *S. cerevisiae* genomes. Nodes present in all accessions were annotated as core, nodes that were only traversed by one accession as private, and all other nodes (>1 and <30 traversals) were classified as soft (shell).

5. Relationship between samples and path/walks

Genomes can contain multiple chromosomes. Depending on genome complexity and size, individual chromosomes may be represented in whole-genome assemblies as single or multiple contigs. To link multiple contigs and/or chromosomes to the same sample, we use PanSN-spec (link here). Similar to GFA walks, sample names are separated by haplotype ID and contig or scaffold name. Walks and paths are interchangeable using PanSN-spec. We work around these samples, which can be thought of as collections of multiple paths or

walks drawn from the same assembly. The number of paths in a single graph is therefore at least as large as the number of samples.

6. More detailed information for some statistics

Similarity/depth

We define similarity by the number of samples traversing a single node. Depth counts the total amount of traversals, regardless of whether it is traversed by a single sample multiple times or different samples. Similarity and depth can be normalized by sample number.

Jumps

Links or edges are jumps from one node to another node. Where node IDs are consecutive integers, we can calculate the difference (in node ID) between two nodes. Large differences normally reflect a link/edge that is not in pan-genomic order and therefore represents an indel. If the node IDs in the path are linearly increasing, there is a high probability that the graph structure is linear too. Node IDs are not required to be in consecutive order, except for jump related statistics, which require a sorted graph. To this end, the command “odgi sort” can be used, which sorts node IDs in pan-genomic order. Learn more about sorting variation graphs here: https://www.biorxiv.org/content/10.1101/2023.09.22.558964v1.

Node degree

Node degree defines the number of edges linking to a single node. We calculate this statistic for all nodes in the graph and compute average, median and standard deviation. Since links/edges are directed in GFA files, we also report “incoming” and “outgoing” node degree separately.

7. Table of all reported features (*gretl* stats)

A table of all reported features can be found in the github repository: https://github.com/MoinSebi/gretl/paper. With future updates, the order of the statistics might change, but the linked table will remain stable to mirror the features represented in this publication.

8. Comparison with other tools

We compared *gretl* with *vg* and *odgi*. We ran vg version v1.54.0 "Parafada" and *odgi* version v0.8 (commit: v0.8.2-92-gbfae0b3). VG was run with the following flags: -z -l -L -s -H -T. We decided to exclude additional flags, since they report features of the graph that might be out of scope for a statistical view of the graph. Detecting and reporting bubbles (variation) of the graph is important, but it does cover an additional layer of the graph, which is out of scope for a fast and accessible statistical check.

For *odgi,* we used the “-m”flag, which internally runs (-S, -W, -L, -b, -l, -g, -s, -f, -d, -p, -N).

9. Supplementary tables and figures

Organism	Chr	Sequences	Samples	Nodes [x1000]	Edges [x1000]	Average node size [bp]
Homo sapiens	**14**	1,882	48	4,155	5,790	65.7
	**18**	1,270	48	2,832	3,980	86.2
	**19**	1,072	48	3,021	4,215	96.2
	**21**	3,029	48	2,761	3,883	99.2
	**22**	1,757	48	3,760	5,224	123.4
Saccharomyces cerevisiae*	**I**	30	30	52	76	9.3
	**III**	30	30	53	73	11.4
	**V**	30	30	125	171	15.9
	**IX**	30	30	67	93	11.8
	**X**	30	30	74	101	11.7
Arabidopsis thaliana**	**1**	67	67	6,891	9,741	17.0
	**2**	67	67	4,927	6,979	15.5
	**3**	67	67	5,977	8,520	15.4
	**4**	67	67	4,747	6,789	18.2
	**5**	67	67	5,657	7,984	19.6

Supplementary Table 1. Information on the genome graphs used in this study.
*Parameter set -p 90, -n 30, -s 5000, -k 31. **Parameter set: -p 90 -s 10000 -G 2000 -n 67 -t 32 -k 49 -P asm5 -O 0.001 -G 700,900,1000.

Name	Metric type	Unit	Range of values	Description	vg	odgi
Paths	Single integer	Dimensionless	All positive	Number of P and W lines in the file		
Samples	Single integer	Dimensionless	All positive	Number of samples. Samples are collections of P/W-lines that are defined by PanSN-spec		
Nodes	Single integer	Dimensionless	All positive	Number of nodes		
Edges	Single integer	Dimensionless	All positive	Number of edges		
N/E ratio	Single number	Dimensionless	All positive	Nodes divided by edges		
Graph size	Single integer	bp	All positive	Total amount of sequence in the graph (um of all nodes) in bp		
Input genome size	Single integer	bp	All positive	Sum of sizes of all input genomes of the graph		
Compression	Single number	Dimensionless	All positive	Graph size divided by input genome size		
Node length	Average, median	bp	All positive	Node length in bp		
Node length top 5%	Average, median	bp	All positive	Average node length of the top 5 % nodes (sorted by size) in bp		
Bin	Single integer	Dimensionless	All positive	Number of nodes in each bin. Bin can be modified by user input		
Similarity	Average, median, std	Dimensionless	All positive	Average similarity of the entire graph		
Similarity (normalized)	Average, median, std	Dimensionless	0-1	Similarity divided by number of samples		
Depth	Average, median, std	Dimensionless	All positive	Average depth of the whole graph		
Depth (normalized)	Average, median, std	Dimensionless	0-1	Depth divided by number of samples		
Node degree	Average, median, std	Dimensionless	All positive	Average node degree. Average number of edges linking to one node (total).		
Inverted edges (normalized)	Average, median	Dimensionless	0-1	Number of edges that change their direction (from + to - or from - to +). Normalized by the total number of edges.		
Negative edges (normalized)	Average, median	Dimensionless	0-1	Number of negative edges. Here, a negative edge is defined to be from "-" to "-". Normalized by the total number of edges.		
Self edges (normalized)	Raw, normalized	Dimensionless	0-1	Number of self edges. Self edges are edges starting and ending at the same node. Normalized by the total number of edges.		
Graph density	Single number	Dimensionless	All positive	Proportion of observed edges and nodes relative to the number of all possible edges. Calculation: Edges / ((Nodes * (Nodes - 1))/2)		

Supplementary Table 2. Graph-centric statistics reported by "*gretl stats*".

Types of values and metric types reported. Green: Reported by other tools. Orange: Can be computed by math operations. Red: Not reported by other tools.

Name	Metric type	Unit	Range of values	Description	vg	odgi
Sequence	average, std	bp	All positive	Total amount of sequence in the path		
Covered	average, std	Dimensionless	All positive	Sequence [bp] / Graph size [bp]		
Nodes	average, std	Dimensionless	All positive	Number of nodes in the path		
Unique edges	average, std	Dimensionless	All positive	Number of unique nodes		
Directed nodes	average, std	Dimensionless	All positive	Number of different directed nodes		
Edges	average, std	Dimensionless	All positive	Number of edges		
Unique Edges	average, std	Dimensionless	All positive	Number of unique edges		
Unique nodes	average, std	Dimensionless	All positive	Number of unique nodes		
Unique nodes	average, std	bp	All positive	Amount of sequence of unique nodes		
Unique nodes (normalized)	average, std	Dimensionless	0-1	Unique nodes / Nodes		
Unique nodes (normalized)	average, std	Dimensionless	0-1	Unique nodes [bp] / Sequence [bp]		
Unique edges (normalized)	average, std	Dimensionless	0-1	Unique Edges / Edges		
Inverted nodes	average, std	Dimensionless	All positive	Number of inverted nodes		
Inverted nodes	average, std	bp	All positive	Amount of sequence of all inverted nodes		
Inverted nodes (normalized)	average, std	Dimensionless	0-1	Inverted nodes / Nodes		
Inverted nodes (normalized)	average, std	Dimensionless	0-1	Inverted nodes [bp] / Sequence [bp]		
Jumps total	average, std	Dimensionless	All positive	Total number of of jumps		
Jumps total (normalized)	average, std	Dimensionless	All positive	Jumps total / Edges		
Jumps larger than X	average, std	Dimensionless	All positive	Number of jumps larger than X		
Jumps larger than X (normalized)	average, std	Dimensionless	0-1	Jumps larger than X / Edges		
Node size average	average, std	Dimensionless	All positive	Average node size		
Node size median	average, std	Dimensionless	All positive	Median node size		
Node size std	average, std	Dimensionless	All positive	Standard deviation of node size		
Depth average	average, std	Dimensionless	All positive	Average depth		
Depth median	average, std	Dimensionless	All positive	Median depth		
Depth std	average, std	Dimensionless	All positive	Standard deviation of depth		
Depth average (normalized)	average, std	Dimensionless	All positive	Depth average / Number of samples		
Depth median (normalized)	average, std	Dimensionless	All positive	Depth median / Number of samples		
Depth std (normalized)	average, std	Dimensionless	All positive	Depth std / Number of samples		
Similarity average	average, std	Dimensionless	All positive	Average similarity		
Similarity median	average, std	Dimensionless	All positive	Median similarity		
Similarity std	average, std	Dimensionless	All positive	Standard deviation of similarity		
Similarity average (normalized)	average, std	Dimensionless	0-1	Similarity average/ Number of samples		
Similarity median (normalized)	average, std	Dimensionless	0-1	Similarity median / Number of samples		
Similarity std (normalized)	average, std	Dimensionless	0-1	Similarity std / Number of samples		
Degree average	average, std	Dimensionless	All positive	Average node degree		
Degree median	average, std	Dimensionless	All positive	Median node degree		

Supplementary Table 3. Path-centric statistics reported by "*gretl stats*". Descriptions for each value and which metric type is reported when run in the default mode. Each value is calculated for the individual path independently and then summarized by the reported metric. Using the "-p" flag reports the independent values for each path separately.

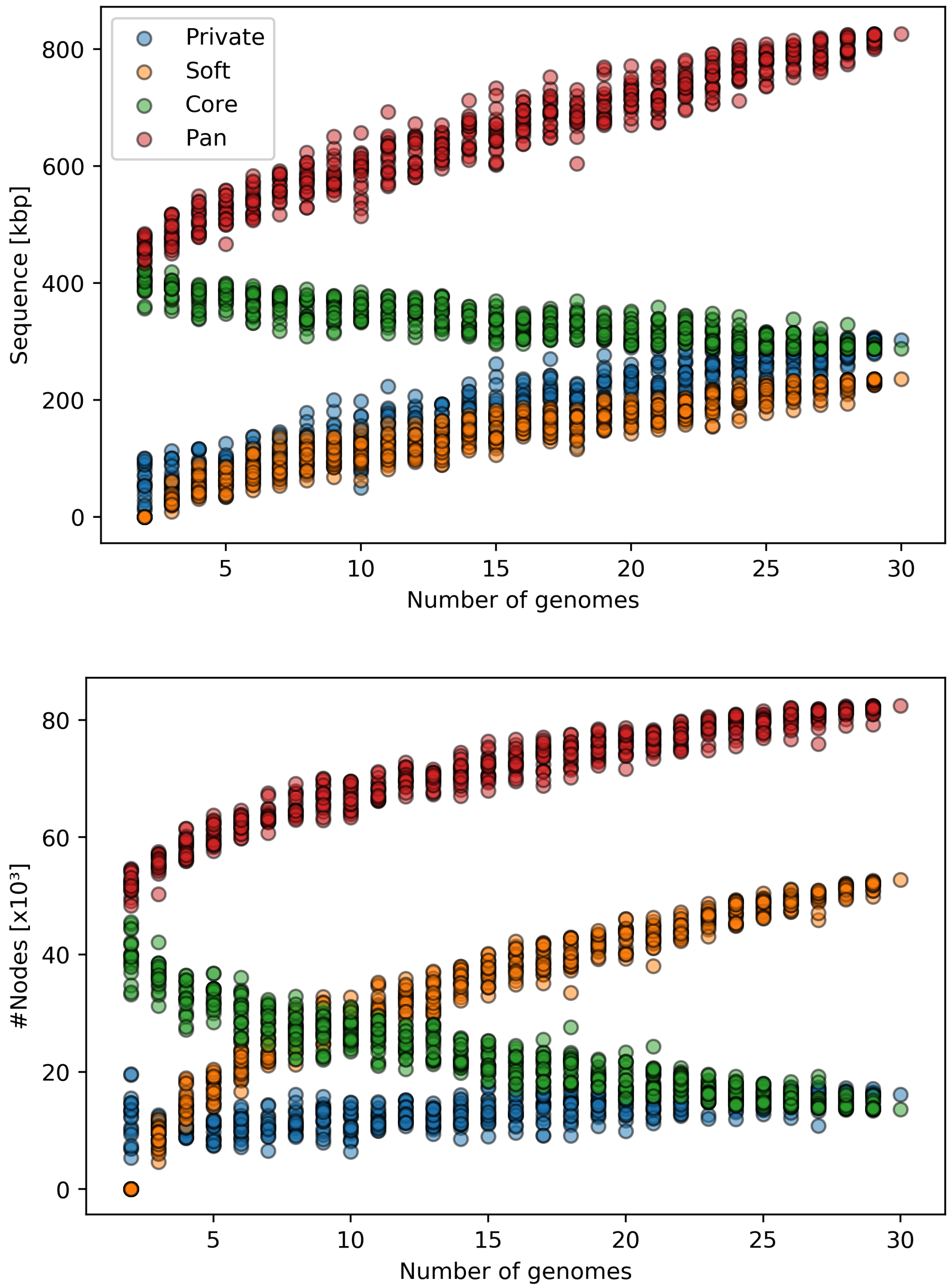

Supplementary Figure 1. Saturation analysis based on the *S. cerevisiae* genome graph for chromosome IX.

Top figure represents the amount of sequence in each category, bottom figure displays the number of nodes across 20 bootstraps**.** Classification as described in Section 4. The pangenome does not seem to saturate, which is mainly due to new variants being added with additional genomes.

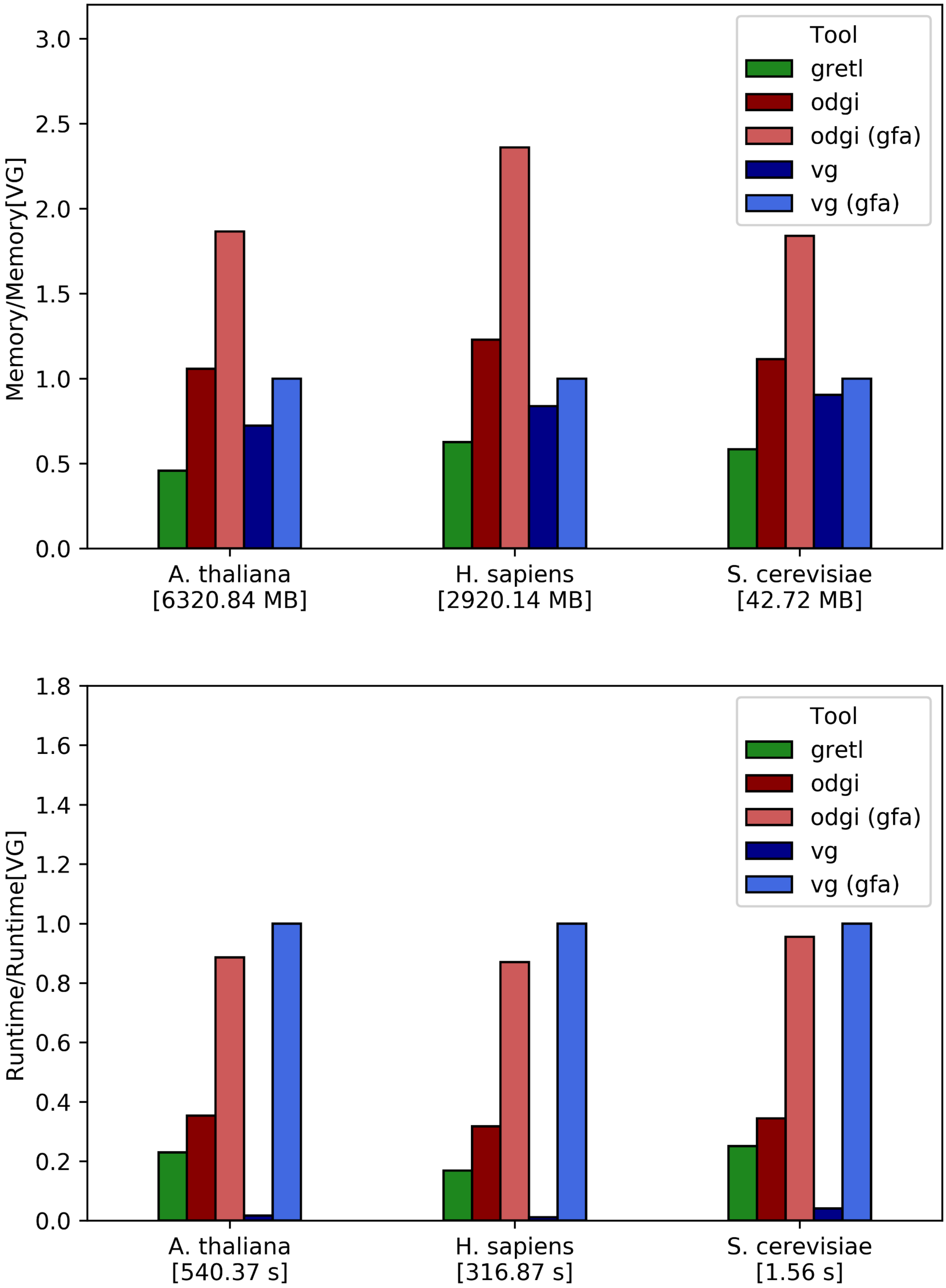

Supplementary Figure 2. Run-time and memory benchmarking for *gretl*, *vg stats* and *odgi stats* in relation to *vg stats*.

We added benchmarks for ***odgi*** and ***vg*** using their exclusive data formats as inputs. ***vg***'s (*gfa*) runtime and memory consumption are indicated in brackets at the bottom.

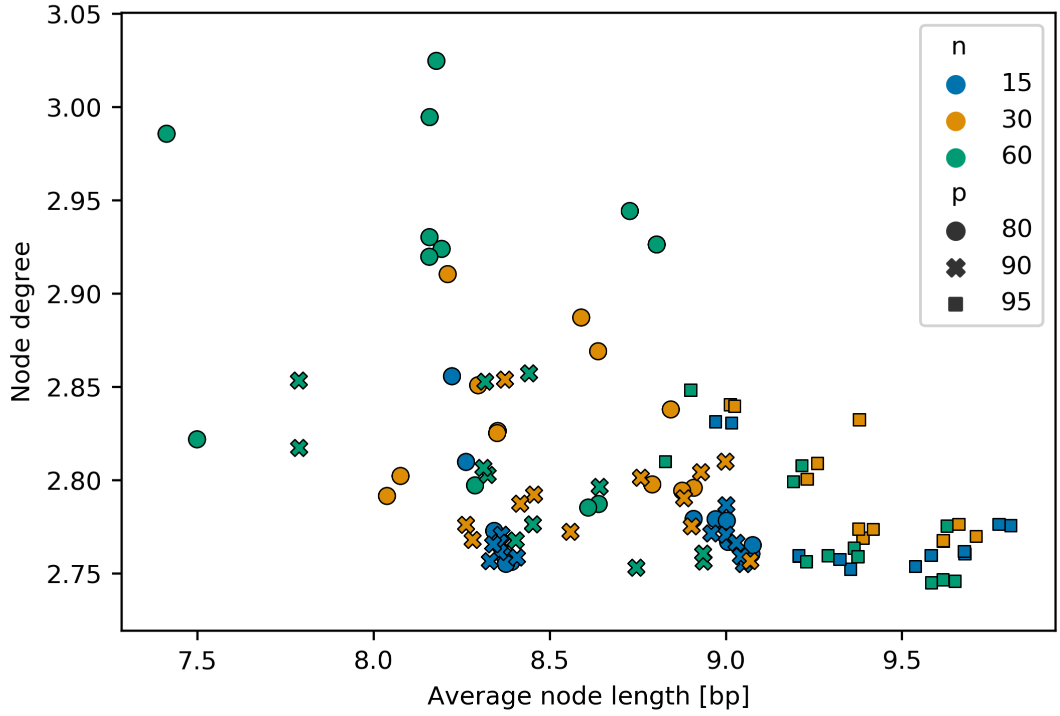

Supplementary Figure 3. Relationship between node degree and the average node length in base pairs for all graphs built with different combinations of parameters. The different colors highlight the “percent identity” in the ***wfmash*** step (-p), the different shapes the (secondary) n-mappings (-n) of ***pggb***. Graphs are based on chromosome IX from 30 *S. cerevisiae* genomes. Graphs built with different “p” can easily be distinguished and this parameter seems to have a strong influence on the graph.

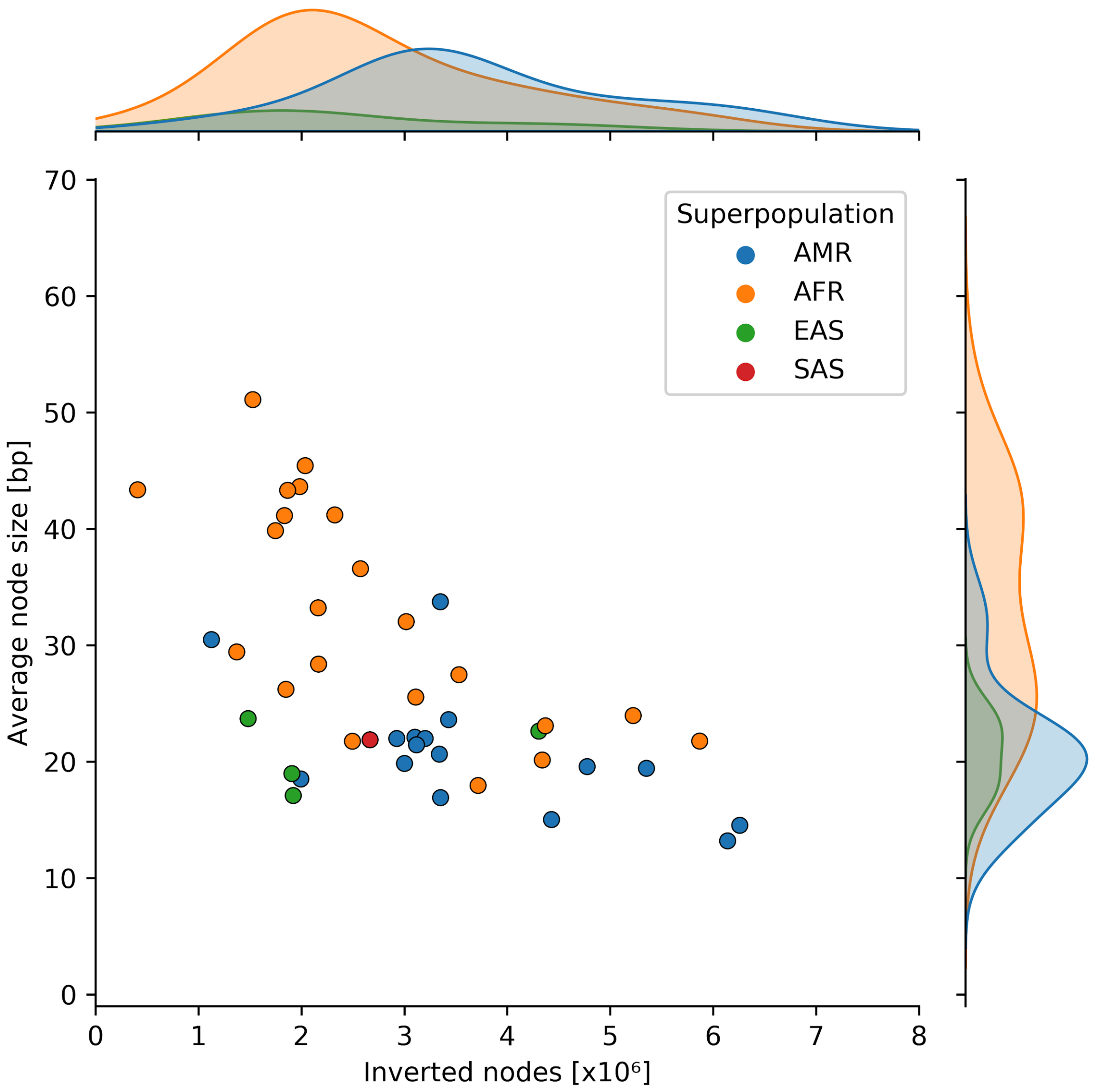

Supplementary Figure 4. Relationship between average node size and the number of inverted nodes of each path in the *H. sapiens* chromosome 18 (hprc) graph.
The path names are annotated to their superpopulation: AMR: Admixed American, AFR: African, EAS: East Asian, SAS: South Asian.

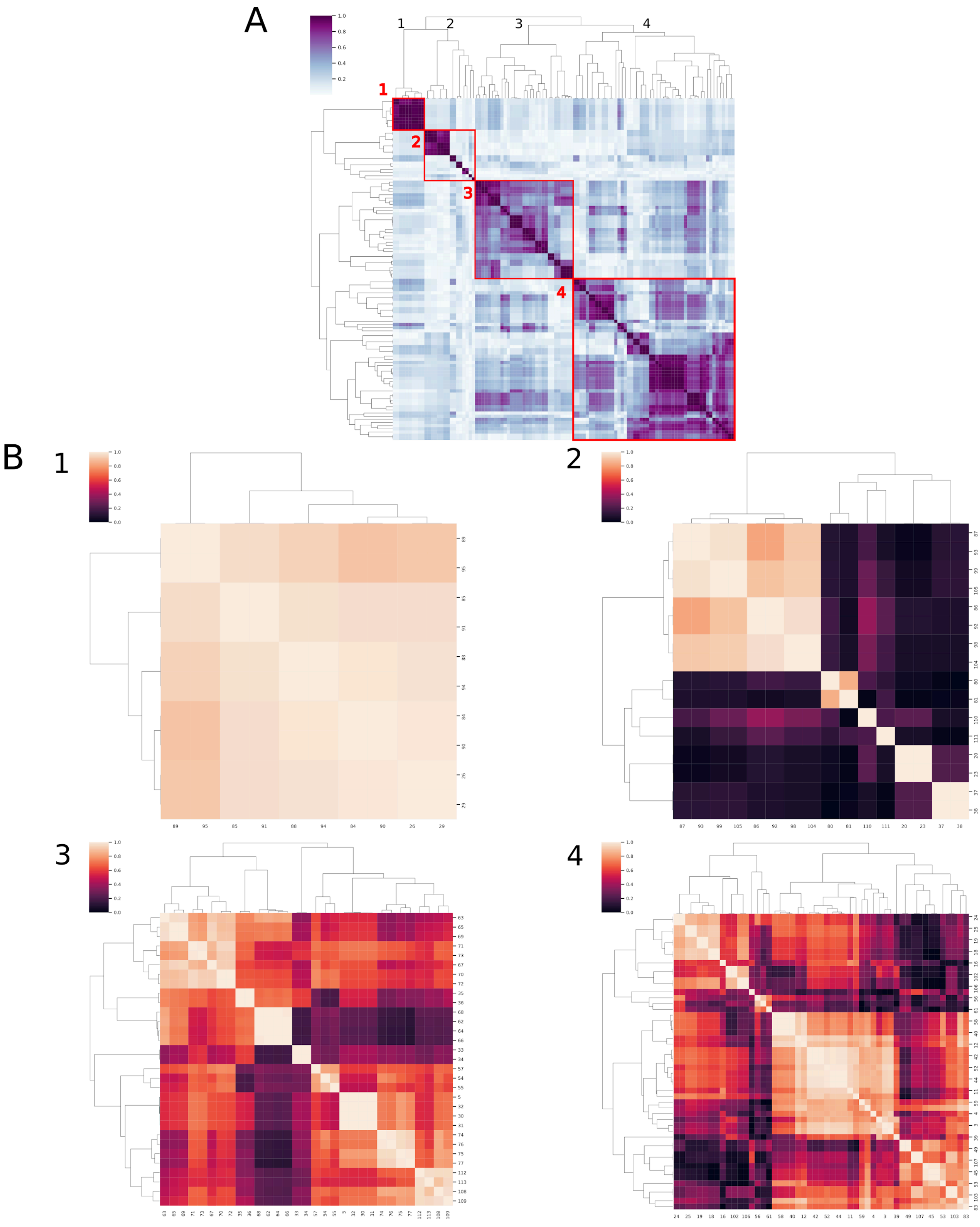

Supplementary Figure 5. Correlation among graph statistics.
Using various graphs constructed from a single dataset but with different parameters, we assessed the correlation among their statistics. The heatmap reveals that numerous statistics are strongly correlated, suggesting that they capture similar features. **A**, Correlation across all statistics. **B**, Close-ups of regions 1-4 from (A), representing the most distinct clusters (clades) based on the hierarchical clustering (method: “average”).

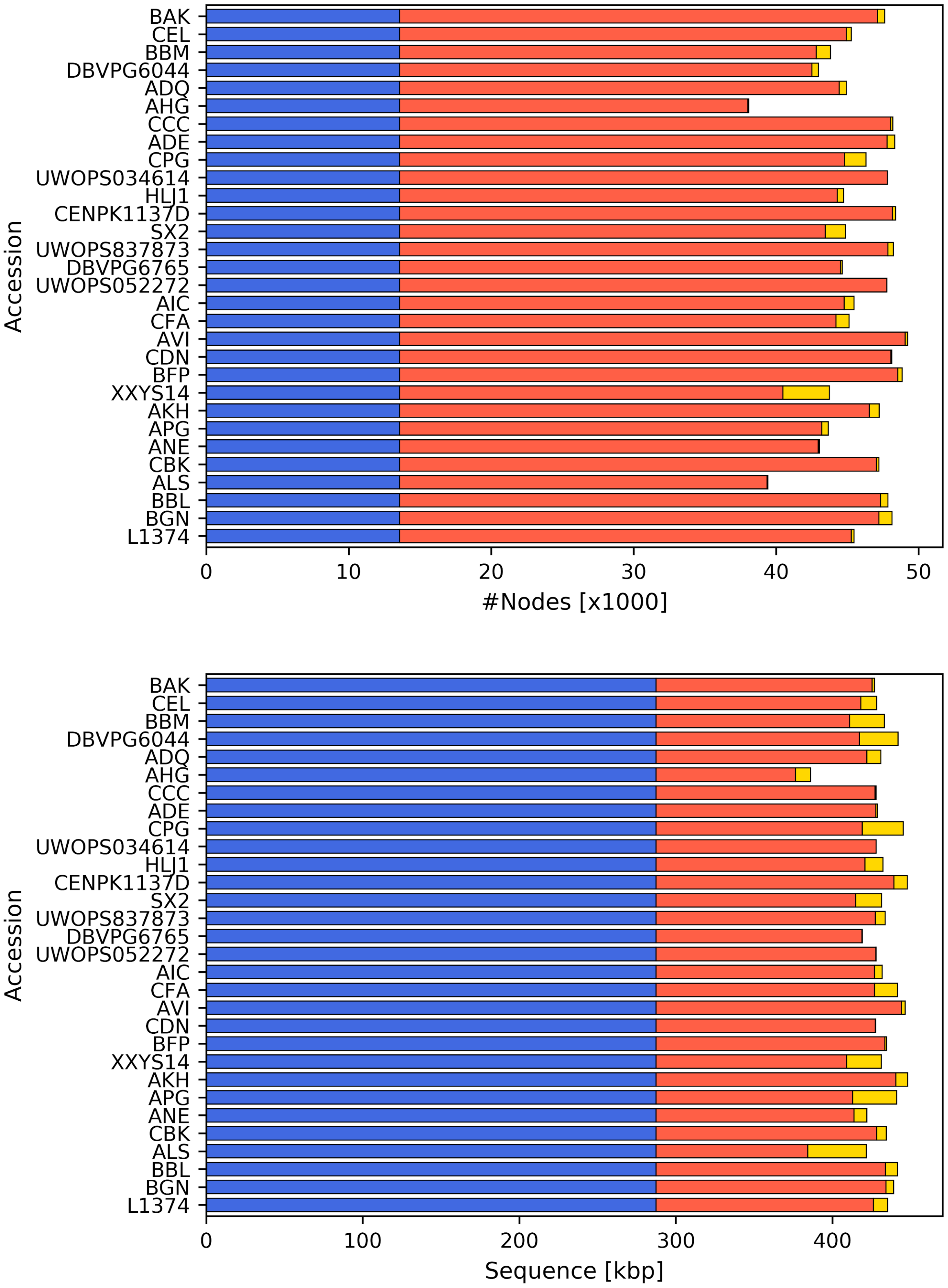

Supplementary Figure 6. ***gretl ps*** **subcommand - Distribution of nodes across various** ***S. cerevisiae*** **genomes.**

See Methods section 1.2.4. Blue: core, orange: soft, yellow: private. Top, number of nodes in each class. Bottom, the amount of sequence for each accession. The genomes (paths) are not in any particular order.

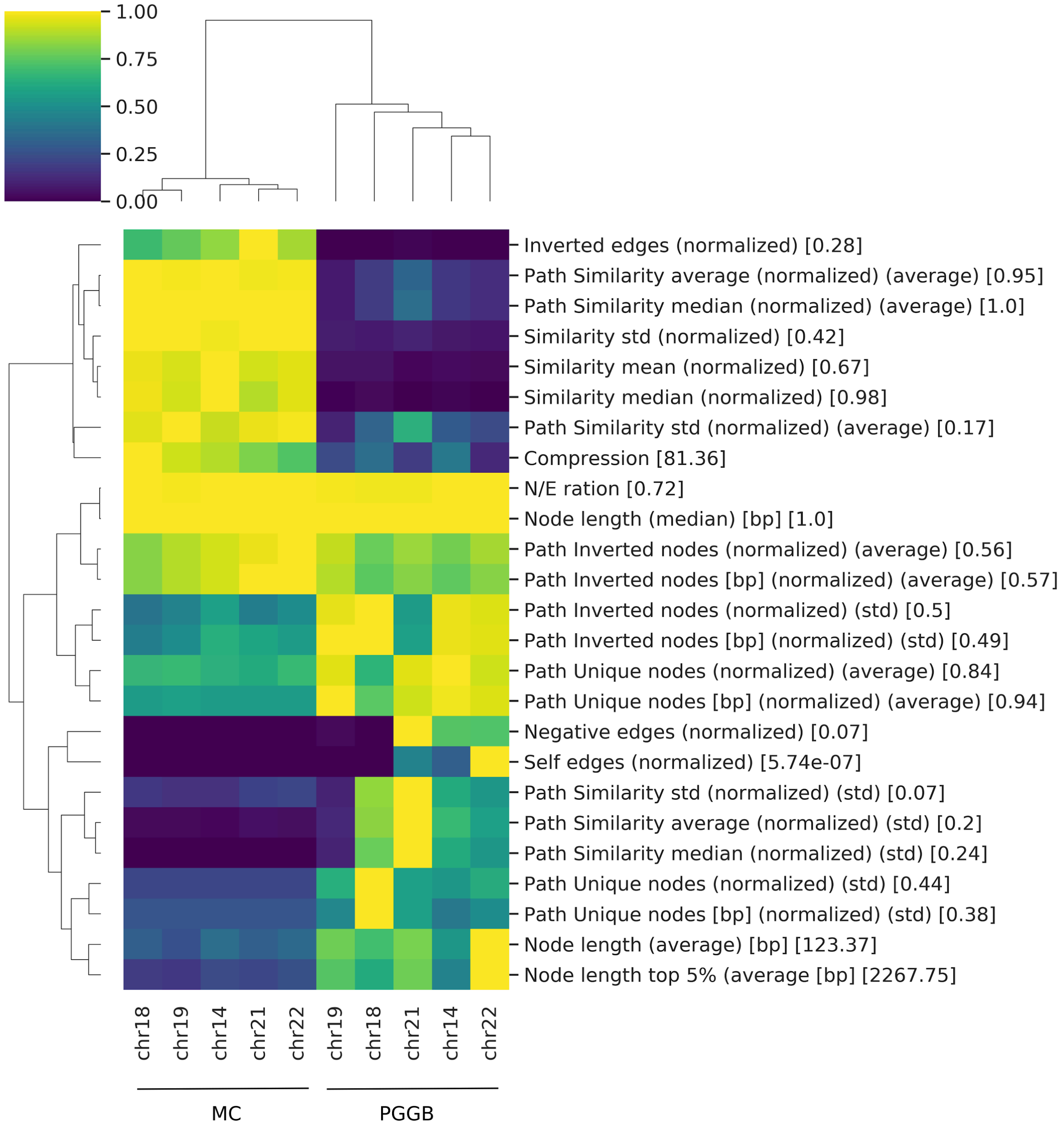

Supplementary Figure 7. ***gretl stats*** **- comparison of different methods.**
Individual graphs cluster by method and not by chromosome. Only ‘normalized’ metrics are shown here. In addition, values were scaled by maximum for each feature (rows). Maximum values shown in brackets.

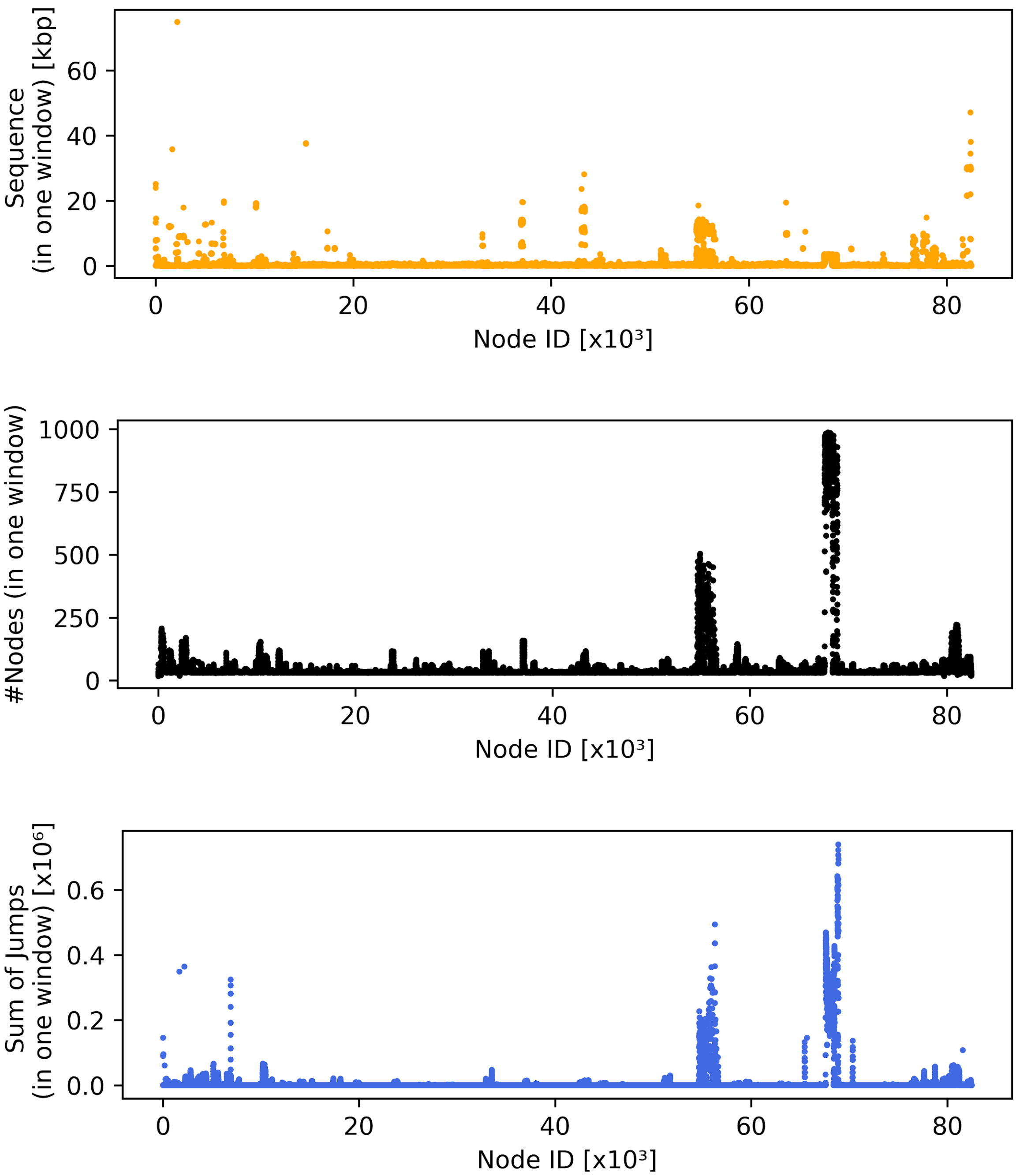

Supplementary Figure 8: *gretl nwindow* - Detection of regions of high local variability. Graph-based window approach iterating over each node in the graph and capturing all nodes in up to 10 steps away. Each "window" is summarized by amount of sequence (top), number of nodes (middle), or summary of node ID distance (jumps, bottom) from the starting node.

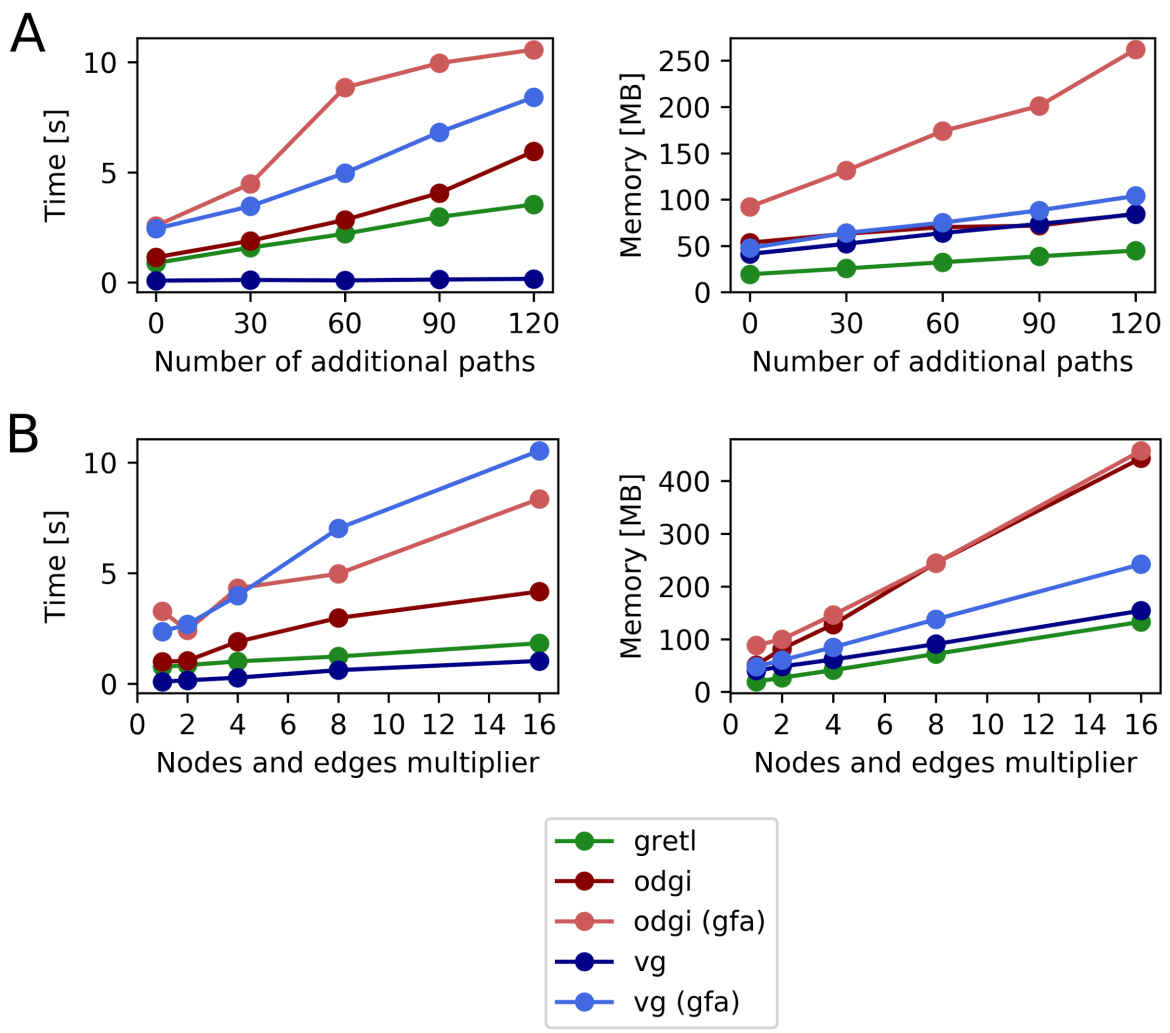

Supplementary Figure 9. Scaling properties.
A, Based on increasing number of paths. **B**, Based on increasing number of nodes/edges. The analyses are based on chromosome V of yeast with 30 paths. The number of paths in (A) was increased in every step by adding all paths from the base graph. For B, we repeatedly (indicated by the multiplier) added the nodes and edges of the base graph to the evaluated graphs. The constant time complexity (A, left) of the ***vg*** tool (on native format) is likely due to not processing path-related information.

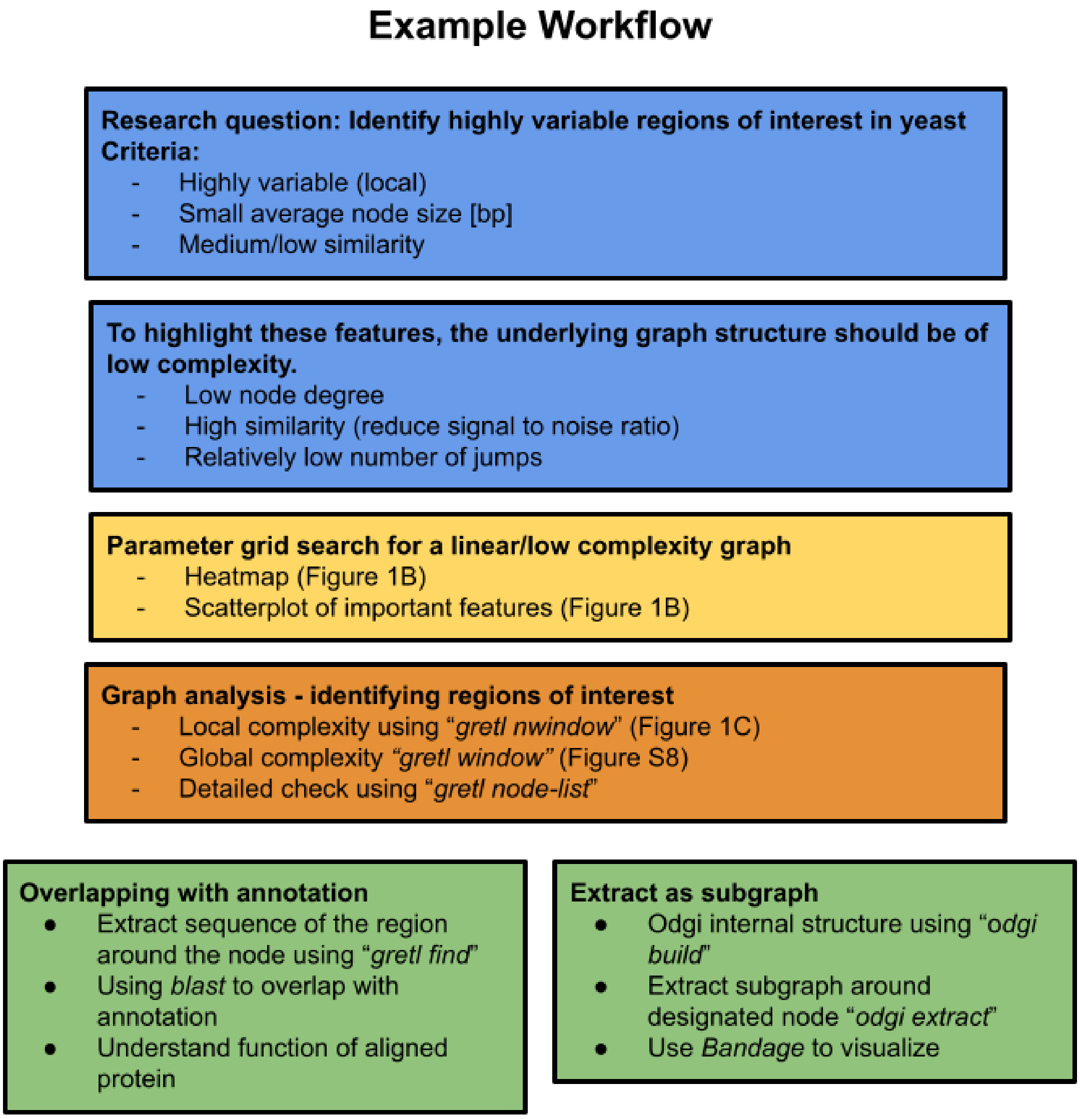

Supplementary Figure 10. Example workflow.
This example had the goal to "Identify highly variable regions based on genome graphs". The target is a specific statistical profile that limits possible candidates for highly variable regions to a reasonable number. After finding the optimal graph (yellow), we continue with several analysis steps (orange). Finally, the regions of interest can be compared to existing annotations, e.g., by BLAST, or the subgraph can be extracted and investigated in detail. A combination of both might be the optimal approach (green).

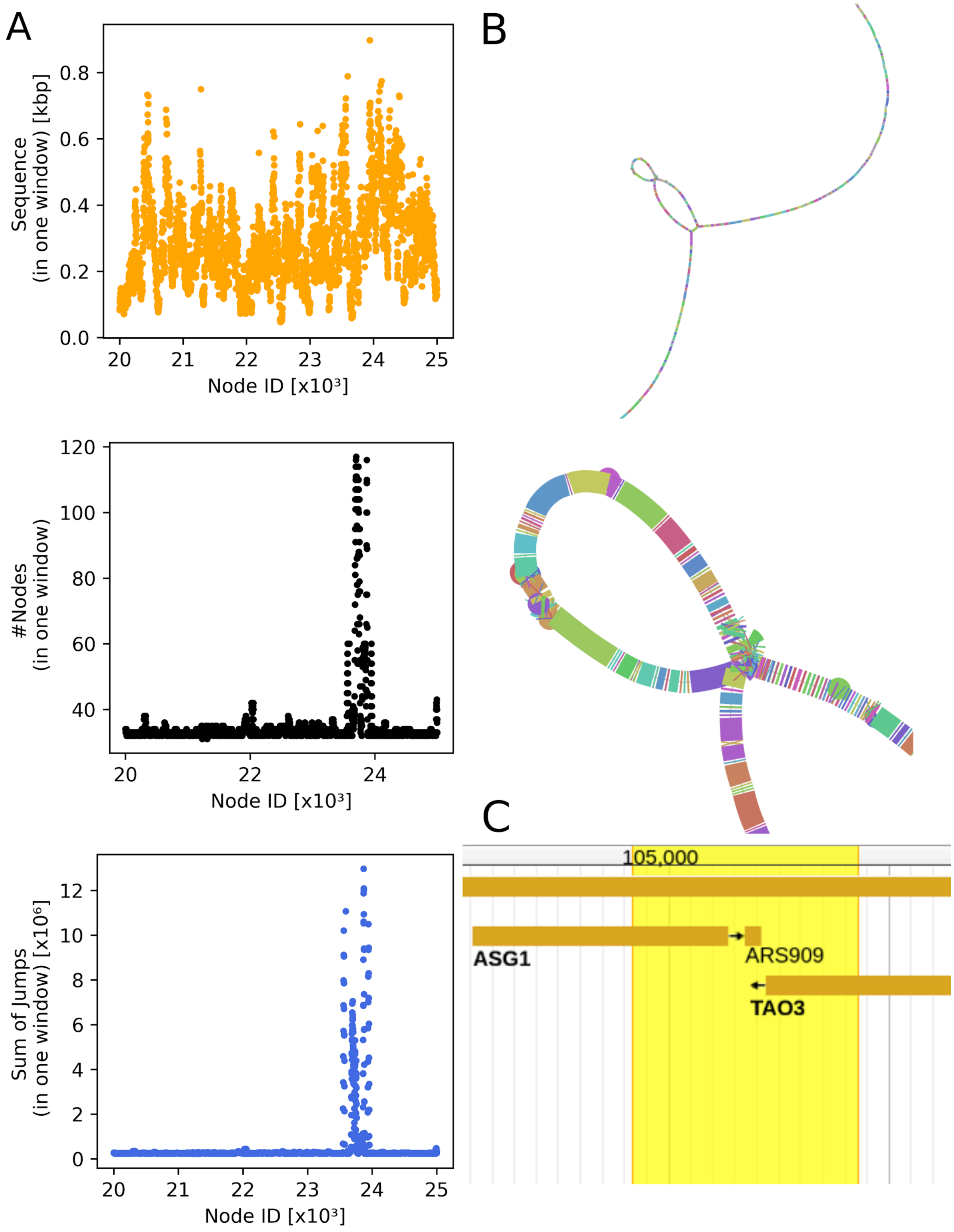

Supplementary Figure 11: Example results.
Here, we identified a double loop structure caused by presence-absence polymorphism resulting in a highly variable region in close proximity to the bifurcation/merging point. **A**, Based on the *nwindow* analysis, nodes with an ID at ~23850 showed a dense pattern with no significant increase in sequence. **B**, The nodes were identified to be in the middle of a double loop structure close to a SNP array, which caused the high number of nodes. **C**, We are not able to comment about the origin of the highly variable region, but from a sequence similarity search with the loop sequence to the reference annotation, we found an autonomously replicating sequence bordered by a regulatory and a signal transduction gene.
